# Nationwide patient registry for GNE myopathy in Japan

**DOI:** 10.1186/s13023-014-0150-4

**Published:** 2014-10-11

**Authors:** Madoka Mori-Yoshimura, Yukiko K Hayashi, Naohiro Yonemoto, Harumasa Nakamura, Miho Murata, Shin’ichi Takeda, Ichizo Nishino, En Kimura

**Affiliations:** Department of Neurology, National Center Hospital, National Center of Neurology and Psychiatry, 4-1-1 Ogawahigashi, Kodaira, Tokyo 187-8551 Japan; Department of Neuromuscular Research, National Institute of Neuroscience, National Center of Neurology and Psychiatry, 4-1-1 Ogawahigashi, Kodaira, Tokyo 187-8502 Japan; Department of Neurophysiology, Tokyo Medical University, 6-1-1 Shinjuku, Shinjuku, Tokyo 160-8402 Japan; Translational Medical Center, National Center of Neurology and Psychiatry, 4-1-1 Ogawahigashi, Kodaira, Tokyo 187-8551 Japan; Department of Molecular Therapy, National Institute of Neuroscience, National Center of Neurology and Psychiatry, 4-1-1 Ogawahigashi, Kodaira, Tokyo 187-8502 Japan

**Keywords:** GNE myopathy, Distal myopathy with rimmed vacuoles (DMRV), Natural history, Remudy, Patient registry

## Abstract

**Background:**

GNE myopathy is a slowly progressive autosomal recessive myopathy caused by mutations in the *GNE* (glucosamine (UDP-N-acetyl)-2-epimerase/N-acetylmannosamine kinase) gene. This study aimed to (1) develop a nationwide patient registry for GNE myopathy in order to facilitate the planning of clinical trials and recruitment of candidates, and (2) gain further insight into the disease for the purpose of improving therapy and care.

**Methods:**

Medical records of genetically-confirmed patients with GNE myopathy at the National Center Hospital of the National Center of Neurology and Psychiatry (NCNP) were retrospectively reviewed in order to obtain data reflecting the severity and progression of the disease. We also referred to items in the datasheet of the nationwide registry of dystrophinopathy patients in the Registry of Muscular Dystrophies (Remudy). Items selected for the registration sheet included age, sex, age at onset, past history and complications, family history, body weight and height, pathological findings of muscle biopsy, grip power, walking ability, respiratory function, cardiac function, willingness to join upcoming clinical trials, and participation in patient associations. A copy of the original genetic analysis report was required of each patient.

**Results:**

We successfully established the Remudy-GNE myopathy. Currently, 121 patients are registered nationwide, and 93 physicians from 73 hospitals collaborated to establish the registry. The mean age at onset was 27.7 ± 9.6 years, and 19.8% (24/121) of patients could walk without assistance. Mean presumed durations from onset to use of assistive devices (cane and/or braces) and a wheelchair, and loss of ambulation were 12.4, 15.2, and 21.1 years, respectively. Three patients had a past history and/or complication of idiopathic thrombocytopenia. To share the progress of this study with the community, newsletters were published on a regular basis, and included information regarding new phase I clinical trials for GNE myopathy. The newsletters also served as a medium to bring attention to the importance of respiratory evaluation and care for respiratory insufficiency.

**Conclusion:**

The Japanese Remudy-GNE myopathy is useful for clarifying the natural history of the disease and recruiting patients with genetically-confirmed GNE myopathy for clinical trials.

**Electronic supplementary material:**

The online version of this article (doi:10.1186/s13023-014-0150-4) contains supplementary material, which is available to authorized users.

## Background

GNE myopathy, also known as distal myopathy with rimmed vacuoles (DMRV), Nonaka myopathy, or hereditary inclusion body myopathy (hIBM), is an early adult-onset myopathy with slow progression that preferentially affects the tibialis anterior muscles and commonly spares the quadriceps femoris muscles [1,2]. GNE myopathy is caused by mutations in the *GNE* gene encoding a bifunctional enzyme [uridine diphosphate-*N*-acetylglucosamine (UDP-GlcNAc) 2-epimerase and N-acetylmannosamine kinase] that catalyzes two rate-limiting reactions in cytosolic sialic acid synthesis [3-7]. Oral sialic acid metabolite supplementation prevents muscle atrophy and weakness in a mouse model of GNE myopathy [8]. While the incidence of GNE myopathy is unknown, more than 200 patients currently exist in Japan [9].

Registries for rare diseases are broadly accepted for their usefulness in obtaining epidemiological data and patient recruitment for clinical trials [10] Translational Research in Europe–Assessment and Treatment of Neuromuscular Diseases (TREAT-MND ALLIANCE), a research network for neuromuscular disorders, developed a global database for patients with Duchenne muscular dystrophy (DMD) [11], spinal muscular atrophy, alpha-dystroglycanopathy with mutations in *FKRP*, and dysferlinopathy [12]. National registries for other muscular dystrophies and myopathies also exist. In 2009, we developed a national registry for neuromuscular diseases (Registry of MUscular DYstrophy; Remudy. http://www.remudy.jp/) in Japan in collaboration with the TREAT-MND ALLIANCE in order to aid in the recruitment of eligible patients for clinical trials, provide information regarding the natural history and epidemiology of diseases, and serve as a source of information on current clinical care [13]. Given that GNE myopathy is quite rare and the fact that clinical trials have already begun on this disease, the establishment of a patient registry is urgently needed, as it would allow for the early recruitment of patients in future clinical trials. Moreover, in addition to contributing to our knowledge on the natural history of GNE myopathy, accurate medical records also serve as a medium to judge clinical trial results. Remudy tentatively registered only male patients with dystrophinopathy. We intend to expand the registry to include patients with GNE myopathy.

Here, we describe the development of a national patient registry for GNE myopathy based on genetic diagnoses, analyze clinical and genetic characteristics of the disease, and provide etiological data important for clinical trials.

## Methods

### Institution, organization, registration method, data collection, and ethical approval

Remudy is supported by Intramural Research Grants (23-4/26-7) for Neurological and Psychiatric Disorders from the National Center of Neurology and Psychiatry (NCNP). Methodology used to establish the Remudy registry system was described previously [11,13,14]. Registry information was provided to interested individuals and their informed consent was obtained. Individuals whose data were included were informed that inclusion in the database confers no obligation to the patient, and that they may be removed from the registry immediately upon request. They were also told that refusal to participate would not affect the patient’s subsequent medical care. This study was approved by the Medical Ethics Committee of the NCNP. Study objectives, design, risks, and benefits of participation were explained to all patients, and their written informed consent was obtained prior to enrollment.

### Patients

Patients can join the registry via three routes: the Remudy homepage, attending specialists of neurology and myology, and patient associations (the Patient Association of Distal Myopathy, PADM; and the Japan Muscular Dystrophy Association, JMDA). This database includes mutation data confirmed by genetic analysis. Prior to launching the registry, members of SOCIETAS NEUOLOGICA JAPONICA (Japanese society of Neurology) were informed about the purpose of the registry, asked to inform their patients about the registry, and to cooperate when patients asked them to confirm medical information regarding the registry through leaflets.

### Structure of the registry form

Based on our review of medical records and prospective natural history studies of GNE myopathy from the National Center Hospital of NCNP and questionnaires from previous studies [15-17], we concluded that walking ability and respiratory function might be important for evaluating disease status. Based on clinical information from patients with GNE and the basic form used in the registry for patients with dystrophinopathy in Remudy [13], we chose items required for registration.

Items in the registry form include past history, complications, family history, disease onset, ambulation status, results of muscle biopsy, and results of genetic analysis. Walking capability, grip power, cardiac and respiratory function, and serum creatinine kinase (CK) levels are also included in GNE myopathy natural history studies, given their relevance to the prognosis as well as their utility as outcome measures. A copy of the original report of the genetic analysis for *GNE* is required for registration, i.e., only patients with a diagnosis confirmed by a genetic report were included in the registry. Participants with only single heterozygous mutations in *GNE* were registered only when they had pathology results indicating the presence of rimmed vacuoles on muscle biopsy.

### Data collection, curation, and accession

All patient data including clinical and genetic information were registered by patients. Each of the attending neurologists filled in information pertaining to past medical history, family history, and data from medical records (biopsy findings, laboratory and physiological data, and information regarding whether the patient had the capacity to understand the study objectives). The patients sent Case Report Forms, along with personal information (mailing address, phone number, and e-mail address), consent to use their information in clinical trials, participation consent for themselves and their attending physicians, and genetic diagnosis. As data were extracted from medical records, this study is a cross-sectional study for the purposes of the present data, and a prospective study for the purposes of the annual data we are currently collecting. After the patient data were registered, medical and genetic curators cleaned up “tentative” data. Clinical curators are neurology specialists in myology at the NCNP, while genetical curator. is a neurologist and myological researcher responsible for genetic diagnosis of GNE myopathy at the NCNP. During the curation process, curators were able to ask the registrants and their attending neurologists to double-check the accuracy of information, with the agreement with both patients and their attending neurologists. As the Remudy-GNE registry utilizes a yearly renewable system to enable prospective data analysis, we asked registrants to renew their data at least once a year and with any change in physical status. All patient data provision is voluntary and data are not shared with any third party without the permission of the committee responsible for information disclosure. The structure of the Case Report Form and required registry items are shown in Table 1.

**Table 1:** **Structure of the Case Report Form and registry items**

Basic information	Date
	Hospital
	ID of hospital
	Name
	Date of birth
	Sex
	Address
	Phone
	E-mail
	Nationality
	Signed up for other registries?
	Attending any clinical trials?
Family history	Family history
	Consanguinity
Diagnosis	Muscle biopsy
	Genetic analysis* facility ( )
	Complications
Patient status	Body weight and height
	Age and symptoms at onset
	Walking capability and wheelchair use
	Grip power
	Respiratory function (VC,%VC, FVC,%FVC, mechanical support)
	Cardiac functions (EF, ES)
	Creatine kinase level

Gray cells are for patients to fill out, and white cells are to be filled out by physicians.

VC: vital capacity, FVC: forced vital capacity, EF: ejection fraction, FS: fraction shortening.

### Medical record analysis

Medical records of all patients with genetically-confirmed GNE myopathy in the National Center Hospital of NCNP were retrospectively reviewed by M. MY.

### Data analysis

Data were summarized using descriptive statistics, including mean, standard deviation (SD), median, range, frequency, and percentage. Each variable was compared using a t*-*test. Spearman’s rank correlation coefficients were used to determine associations between variables. Time from disease onset to walking with assistance, time from disease onset to wheelchair use, and time from disease onset to loss of ambulation were evaluated using the Kaplan–Meier method. All statistical analyses were performed using SPSS for Macintosh (Version 18; SPSS Inc., Chicago, IL).

## Results

### General characteristics at study entry

**Table 2:** **Participant characteristics**

		**n**	**%**	**Mean ± SD**	**Minimum**	**Median**	**Maximum**
Age		121	100	44.8 ± 13.0	21	43	85
Sex	Male	55	44				
	Female	66	55				
Age at onset		121	100	27.7 ± 9.6	15	26	61
Body mass index		121	100	21.4 ± 4.4	12.1	21	37.8
	Normal	65	54				
	Underweight	36	30				
	Obesity	20	16				
Walking capability	Ambulant without assistance	24	20				
	Ambulant with assistance	45	37				
	Non-ambulant	52	43				
Wheelchair use	Never	43	36				
	Part-time	28	23				
	Full time	50	41				
Respiratory function (%FVC)	Performed	79	65	88.4 ± 23.7	16.4	92.8	130
	Not performed	42	35				
	Normal (FVC ≥ 80%)	26	33*				
	Decreased	53	67*				
	Nocturnal NPPV	2	2				
Cardiac function	Performed	35	29				
(EF, ES)	Not performed	86	71				
	Normal	35	100*				
	Decreased	0	0*				
Grip power (kg)	Performed	96	79	5.5 ± 7.2	0	2.9	30
	Not performed	25	21				
	Grip power = 0	36	38*				
CK (Iu/L)	Performed	120	99	357.8 ± 209.0	11	209	459
	Not performed	1	1				

*ratio of examined/performed.

**Figure 1:**
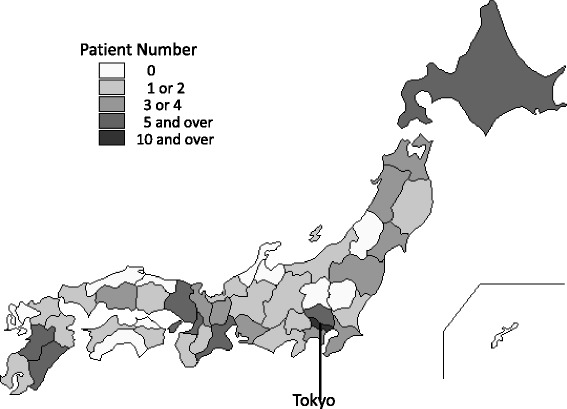
**Participant distribution.** Participants were distributed throughout Japan (38/47 prefectures), and 92 physicians in 73 institutes agreed to contribute to the registry.

As of the end of October 2013, a total of 121 Japanese participants with GNE myopathy (55 men and 66 women) had registered (Table 2). Mean ages at data collection and disease onset were 44.9 ± 13.2 years (mean ± SD) (median, 43 years; range, 21–85 years) and 27.9 ± 9.6 years (median, 26 years; range, 12–61 years), respectively. The registry included participants from throughout Japan (38/47 prefectures) who were recruited through a collaboration with 92 attending physicians from 73 institutes (Figure 1). Among the 52 genetically-confirmed patients with GNE myopathy who had visited NCNP, 32 (62%) participated in the patient registry.

### GNE mutations

Thirty-nine of 121 participants (32.3%) harbored a homozygous mutation in *GNE* and 64.5% (78/121) had a compound heterozygous mutation. Only single heterozygous mutations were found in four (3.3%) participants (Additional file 1: Table S1). Among participants with a homozygous mutation, 82% (32/39), 8% (3/39), and 5% (2/39) harbored p. V572L, p. C13S, and p. M712T mutations, respectively. Homozygous mutations of p. D176V and A630T were identified in only one participant.

Of those carrying two heterozygous mutations, 31% (24/78) had p. D176V/p. V572L mutations, while the remaining participants carried other combinations of mutations. The frequency of the p. V572L mutation was 46% (106/230), p. D176V was 25% (58/230), p. C13S was 4% (9/230), and each of p. M712T and p. A631V was 2% (4/230) (Additional file 2: Table S2). One patient with a single heterozygous mutation visited the NCNP Hospital, so we reviewed his medical records and noted that he was showing clinical symptoms of GNE myopathy as well as pathological features.

### Family history

Thirty-nine of 121 participants (32.2%) had a family history of GNE myopathy. Eleven of 121 (9.1%) were from consanguineous parents. Among the 39 participants with homozygous mutations, 9 (23.1%) had consanguineous parents; 2 participants with a compound heterozygous mutation were from one family, as their mothers and fathers were siblings (i.e., these participants were double cousins).

### Complications and past medical history

A detailed review of medical histories revealed that three participants had hypertension, two had diabetes mellitus, and two had hyperlipidemia. Two participants were diagnosed with obstructive sleep apnea syndrome, one of whom required continuous positive airway pressure. Atopic dermatitis and mastopathy were seen in one participant each. Of note, three participants had a past history of idiopathic thrombocytopenia (ITP). We obtained additional medical histories for three patients with histories of ITP. All three had experienced bleeding symptoms and had undergone intravenous and/or oral steroid therapy. Two of them were hospitalized for this therapy. Patients were unable to recall the platelet count or platelet-associated IgG (PAIgG). However, two patients presented with low platelet counts, one of whom was PAIgG-positive at the time of registration.

To clarify whether patients with GNE myopathy had thrombocytopenia, we reviewed blood counts of those with genetically-confirmed GNE myopathy. Among 52 patients with GNE myopathy in NCNP (including the three participants with a past history of ITP), mean platelet counts were 22.1 × 10^4^/μl (normal range: 15–35 × 10^4^/μl). Importantly, three patients, including two with a past history of ITP, had decreased platelet counts of 9.5, 10.3, and 7.1 × 10^4^/μl, and carried *GNE* mutations of p. R420X/ p. V572L, 383insT/ p. V572L, and p. R8X/p. V572L.

### Onset and ambulation status

**Figure 2:**
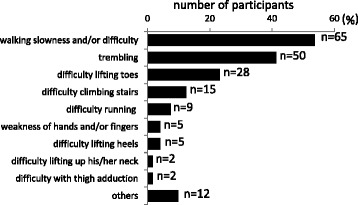
**Initial symptoms.** The most common initial symptom was walking slowness and/or difficulty (54%). Difficulty lifting toes (23%) due to foot drop was the third most common symptom, whereas certain populations had difficulty lifting heels (4%). Some participants had weakness in the hands and/or fingers (4%) and difficulty lifting the neck (2%) from the time of disease onset. The number of participants with the indicated symptoms are shown.

Mean age at disease onset for the 121 registered participants was 27.7 ± 9.6 years (median, 27.5 years; interquartile range, 15–61). Initial symptoms were walking slowness and/or difficulty (65/121, 54%), stumbling (50/121, 41%), difficulty lifting toes (28/121, 23%), difficulty climbing stairs (12/121, 12%), difficulty running (9/121, 7%), difficulty lifting heels with a weakness of hands and/or fingers (5/121, 4%), and difficulty in thigh adduction and lifting the neck (2/121, 2%) (Figure 2). As weakness in the anterior parts is thought to be more prominent than that in the posterior calf in GNE myopathy, we reviewed medical records of the 62 patients who received treatment at NCNP hospitals and identified two patients for whom the first symptom was “difficulty lifting heels.” Prominent calf weakness (MMT ankle dorsoflexion 5, plantar flexion 2) was evident in these patients, along with marked fat replacement in the calf muscles (Additional file 3: Figure S1).

Table 2 summarizes the clinical characteristics of participants included in the registry. A total of 20% (24/121) of participants were ambulant without assistance, 37% (45/121) required assistance (e.g., canes and/or braces), and 43% (52/121) had lost ambulation. Mean age at loss of ambulation was 35.4 ± 11.3 years. Kaplan–Meier analysis revealed a median time from disease onset to walking with assistance of 8.9 years (95%CI, 6.3-9.7), from disease onset to wheelchair use of 14.0 years (95%CI, 11.8-16.2), and from disease onset to loss of ambulation of 21.0 years (95%CI, 15.4-26.6) (Table 3).

**Table 3:** **Analysis of time from disease onset to walking with assistance, wheelchair use, and ambulation loss**

									**Incidence-free proportion**	
	**Mean**	**SD**	**95% CI**		**Median**	**Interquartile range**	**95% CI**		**10 years**	**20 years**
Aassistive device use	12.4	1.3	10.0	14.9	8.0	5.0-13.0	6.3	9.7	0.38	0.18
Wheelchair use	15.2	0.8	13.6	16.9	14.0	8.0-22.0	11.8	16.2	0.64	0.29
Loss of ambulation	21.1	1.4	18.3	23.8	21.0	11.0-37.0	15.4	26.6	0.78	0.51

### Body Mass Index (BMI)

**Figure 3:**
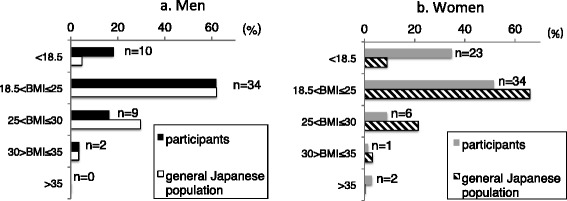
**BMI of participants and the general adult Japanese population (aged >20 years).** More registry participants were underweight compared to the general population. Proportions of underweight participants were 18.2% and 34.8% for men and women, respectively, and 4.7% and 9.1% for healthy men and women, respectively. There were fewer obese participants relative to the general population. **a**. black column: participants, open column: general Japanese population. **b**. gray column: participants, diagonal column: general Japanese population.

BMI of 65/121 (54%) participants were within the normal range in Japan (18.5-25) [13], whereas 36/121 (30%) were under the normal range and 20/121 (16%) were obese. Among the 20 obese participants, two were severely obese (>35%) by Japanese standards [18]. Mean BMI of non-ambulant participants was higher than that of ambulant participants, although the difference was not significant (non-ambulant 22.0 ± 4.3 vs. ambulant 20.6 ± 4.6, p = 0.077). The number of participants who were underweight was greater than that of the normal population. Proportions of men and women who were underweight were 18.2% (n = 11; 16.4 ± 1.9; median, 17.2; range, 12.1-18.5) and 34.8% (n = 23; 16.9 ± 1.3; median, 17.1; range, 13.6-18.4), respectively, and were 4.7% and 9.1% among healthy men and women, respectively. There were fewer obese participants compared to the normal population (Figure 3) [18]. We identified no significant correlations between BMI and other items, with the exception of age (r = 0.291, p = 0.001).

### Cardiopulmonary function

Information on pulmonary and cardiac function was available for 65% (79/121) and 34% (41/121) of participants, respectively. Of those examined, 33% (26/79) had respiratory dysfunction [% forced vital capacity (%FVC < 80)], and two were using nocturnal non-invasive positive pressure ventilation (NPPV).%FVC was significantly correlated with disease duration (ρ = 0.479, p < 0.01) and serum CK levels (ρ = 0.573, p < 0.01). None of the participants who underwent ultrasound cardiographic examination had cardiac dysfunction (ejection fraction, 50-82%; fraction shortening (FS), 25-50%). Mean serum CK level was 459.1 ± 355.0 IU/L (median, 202; range, 11–3133).

### Bulletin, newsletter, and facilitation of participant recruitment through GNE myopathy registry

We have been publishing bulletins every three months and sending them to participants and doctors who join Remudy. The bulletin includes useful information regarding clinical care, translational medicine, and clinical trials, as well as articles introducing specialists and specialized hospitals for muscle diseases. These contents are also available on the Remudy homepage. Participant recruitment has also started for additional phase I clinical trials via the Remudy GNE myopathy registry homepage [19].

## Discussion

To our knowledge, we describe the first patient registry for GNE myopathy in the world. This registry will contribute to the analysis of the natural history of GNE myopathy and aid in the recruitment of participants for clinical trials.

Participants with GNE myopathy were widely distributed throughout Japan, with 1.7 patients per hospital and 1.3 patients per physician in this study. In contrast, there were 5.8 patients with dystrophinopathy (60% of patients with DMD) per hospital and 3.6 per physician in the dystrophinopathy registry. Thus, while patients with GNE myopathy appeared to be dispersed throughout Japan, patients with dystrophinopathy were concentrated in specialized hospitals, given the need for cardiopulmonary care. This indicates that Remudy may serve a very important role in disseminating clinical information to patients with GNE myopathy and their doctors who are dispersed throughout Japan. The patient registry is also useful in that it allows for recruiting patients and resolving data deviation in comparison with analyses by isolated institutions. For example, the age at disease onset in the Remudy-GNE cohort was later than that determined from an analysis of medical records at the NCNP Hospital (26.8 ± 9.0 years). In our previous questionnaire-based study of core muscle disease center patients, we reported a median proportional duration from disease onset to walking with assistance, wheelchair use, and loss of ambulation of 7.0 ± 0.4 years, 11.5 ± 1.2 years, and 17.0 ± 2.1 years, respectively [14], which were all shorter than the durations determined in the present study. We speculate that this discrepancy may reflect the more advanced disease status of patients at neuromuscular disease-specialized center hospitals. Future improvement of Remudy-GNE registry may conclude why these bias were found in this study.

Three (2.5%) of 121 participants had a past history of ITP in our cohort. As the total number of patients with ITP is estimated to be 20,000 in Japan, with an annual occurrence of 3,000 [20], and the Japanese population was 1.27 × 10^8^ in 2013, the prevalence of ITP is expected to be 15.7 per 100,000 (1.57 × 10^−2^%). This means that the frequency of ITP among patients with GNE myopathy is 158 times higher than the general population, at least in our cohort.

UDP-GlcNAc 2-epimerase is a major determinant of cell surface sialylation in human hematopoietic cell lines and a critical regulator of the function of specific cell surface adhesion molecules [6]. Thus, alterations in platelets may occur in patients with GNE myopathy. For example, platelets from patients with ITP show increased electrophoretic mobility, reflecting increased sialic acid content [21]. Among the three participants with decreased platelet counts, two had ITP, raising the possibility of a causal relationship between GNE mutations and ITP, although the underlying mechanisms are unclear and further studies would be necessary to address this issue. Of note, however, this information was obtained based on self-report by patients and/or their families. Thus, the accuracy of the diagnosis is unclear.

It is noteworthy that in some patients, initial symptoms were difficulty lifting heels, but not toes. It is conventionally thought that the initial symptom of GNE myopathy is “foot drop,” as tibialis anterior muscles are strikingly affected. Our study suggests that patients whose symptoms start with calf muscle weakness may have GNE myopathy. It is also surprising that some patients had neck and finger weakness from disease onset, despite GNE myopathy being known as “distal myopathy.” Thus, GNE myopathy appears to be associated with more phenotypes than expected. However, we are not confident that all patients who chose “difficulty lifting heels” exhibited prominent calf weakness in reality as well, i.e., they experienced greater calf weakness relative to tibialis anterior muscle weakness. This is one limitation of using medical histories and a registration system to collect patient data.

Although we previously reported respiratory dysfunction associated with GNE myopathy [15], 35% of participants in the present study were not examined for respiratory function, indicating that many physicians and neurologists are unaware of the clinical significance of respiratory function in the context of this disease. Although we did not observe any cases of cardiac dysfunction, it may occur in older patients or those with advanced disease. Supporting this is evidence from a study showing that 20% of GNE myopathy mice develop fibrosis in cardiac tissue after 30 weeks of age, with some exhibiting marked endomysial fibrosis, amyloid deposition, and occasionally rimmed vacuoles in cardiomyocytes [8]. This suggests that the risk of cardiopulmonary dysfunction in GNE myopathy should be considered.

In this study, four participants harbored single heterozygous mutations, although they exhibited clinicopathologically definite findings of GNE myopathy. The age at disease onset did not significantly differ between homozygotes and compound heterozygotes. Given that we limited our analysis to all exons and their flanking introns, it is possible that single heterozygotes who exhibited features of GNE myopathy may have mutations in other genomic regions of *GNE*. Yet, in the absence of data using disease-specific biomarkers, it is difficult to distinguish whether these participants had other myopathies and carried a single heterozygous mutation in the *GNE* gene.

Among registry participants, 46% were in the abnormal range of BMI, and the number of underweight participants was markedly higher in both men and women, compared to the normal population. None of the participants or patients of NCNP had dysphagia or other medical problems which might promote weight loss. The BMI of non-ambulant patients tended to be higher than ambulant patients, suggesting that muscle atrophy itself did not cause weight loss. Mechanisms underlying the weight changes may differ from those observed in muscular dystrophy such as DMD [22] or myotonic dystrophy [23], given that obesity was not an issue with most patients with GNE myopathy. It is not clear whether being underweight is beneficial relative to having normal weight in these patients. Prospective analyses will be needed to reveal the relationship between motor function prognosis and body weight.

This study has some limitations worth noting. First, we could not unify the method of grip power assessment. Second, we relied on descriptions of motor function as a crude benchmark for designing clinical trials. Finally, we could not address phenotype-genotype correlations in more depth than was previously reported [9], given the limited number of homozygote patients harboring mutations other than V572L. A larger cohort will be needed to address genotype-phenotype correlations. Similar to our collaborations involving the dystrophinopathy registry, we are currently in discussions to harmonize the international registry of GNE myopathy of the TREAT-NMD ALLIANCE [ClinicalTrials.gov Identifier NCT01784679, http://www.treat-nmd.eu/gne/patient-registries/international-registry/] (GNE-DMP), in hopes of gaining further insights into the disease. There are two major differences between GNE-DMP. First, as the Remudy aims to establish registration according to genetic diagnosis, inclusion criteria for genetics-based longitudinal natural history studies employing the Remudy-GNE registry require genetic diagnosis (including single heterozygote). Second, we are the only Japanese language registry system. Japan has one of the largest patient groups with GNE myopathy in the world [24]. It is important that patients with this disease receive information in their native language, and that domestic information is supplied for the purpose of Japanese patient accession. Harmonisation would be conducted in order to avoid duplication and double registration of GNE patients while providing the same benefits and opportunities to patients, regardless of where they live. Both registries are similar in their processes utilized for data collection as well as their fundamental ideas regarding the registries, and thus we hope to merge the two registries at some point. According to a tentative agreement, the Remudy-GNE will remain the primary entryway into the international registry as well as serve as the contact site for Japanese patients, and only anonymous data will be stored in the joint data set. Strategies for merging the two registries are currently under consideration.

Our Japanese registry and the TREAT-MND ALLIANCE registry work in close collaboration, and will serve as irreplaceable infrastructures that accelerate research, therapy development, and trial readiness, in addition to increasing opportunities for collaboration and improving global patient care.

## Conclusion

The patient registry for GNE myopathy in Japan is useful for gaining a better understanding of the disease, and recruiting patients with genetically-confirmed GNE myopathy for upcoming clinical trials. Further advances and insights can be expected through a soon-to-be-launched international GNE myopathy registry.

## Additional files

Additional file 1: Table S1. Genotyping. ED = Glucosamine (UDP-N-acetyl)-2-epimerase domain, KD = N-acetylmannosamine kinase domain. There were more participants who were either homozygous for p.V572L or heterozygous for p.D176V/p.V572L compared to those with other mutations. Although p.D176V was the second most frequent mutation, there was only one participant in the registry who was homozygous for p.D176V. Additional file 2: Table S2. Allelic frequency. p.V572L was the most frequent mutation. Additional file 3: Figure S1. Muscle CT of 29 year-old GNE myopathy patient who reported difficulty lifting his heels as one of the first symptoms. Ankle plantar flexion (MMT 2) was prominently impaired (MMT5), and muscle CT revealed that fatty replacement and atrophy were far more prominent in the calf than the anterior part of the lower legs.

## Abbreviations

GNE: Glucosamine (UDP-N-acetyl)-2-epimerase/N-acetylmannosamine kinase
NCNP: National Center of Neurology and Psychiatry
REMUDY: Registry of muscular dystrophies
DMRV: Distal myopathy with rimmed vacuoles
UDP-GlcNAc: Uridine diphosphate-*N*-acetylglucosamine
TREAT-MND ALLIANCE: Translational Research in Europe–Assessment and Treatment of Neuromuscular Diseases
SD: Standard deviation
ITP: Idiopathic thrombocytopenia
FVC: Forced vital capacity
NPPV: Non-invasive positive pressure ventilation
EF: Ejection fraction
FS: Fraction shortening
CK: Creatine kinase
