# Supplementary figures and images for: Nationwide patient registry for GNE myopathy in Japan
Source: Orphanet J Rare Dis. 2014 Oct 11;9:150. doi: 10.1186/s13023-014-0150-4 (PMC4203883; doi:10.1186/s13023-014-0150-4)

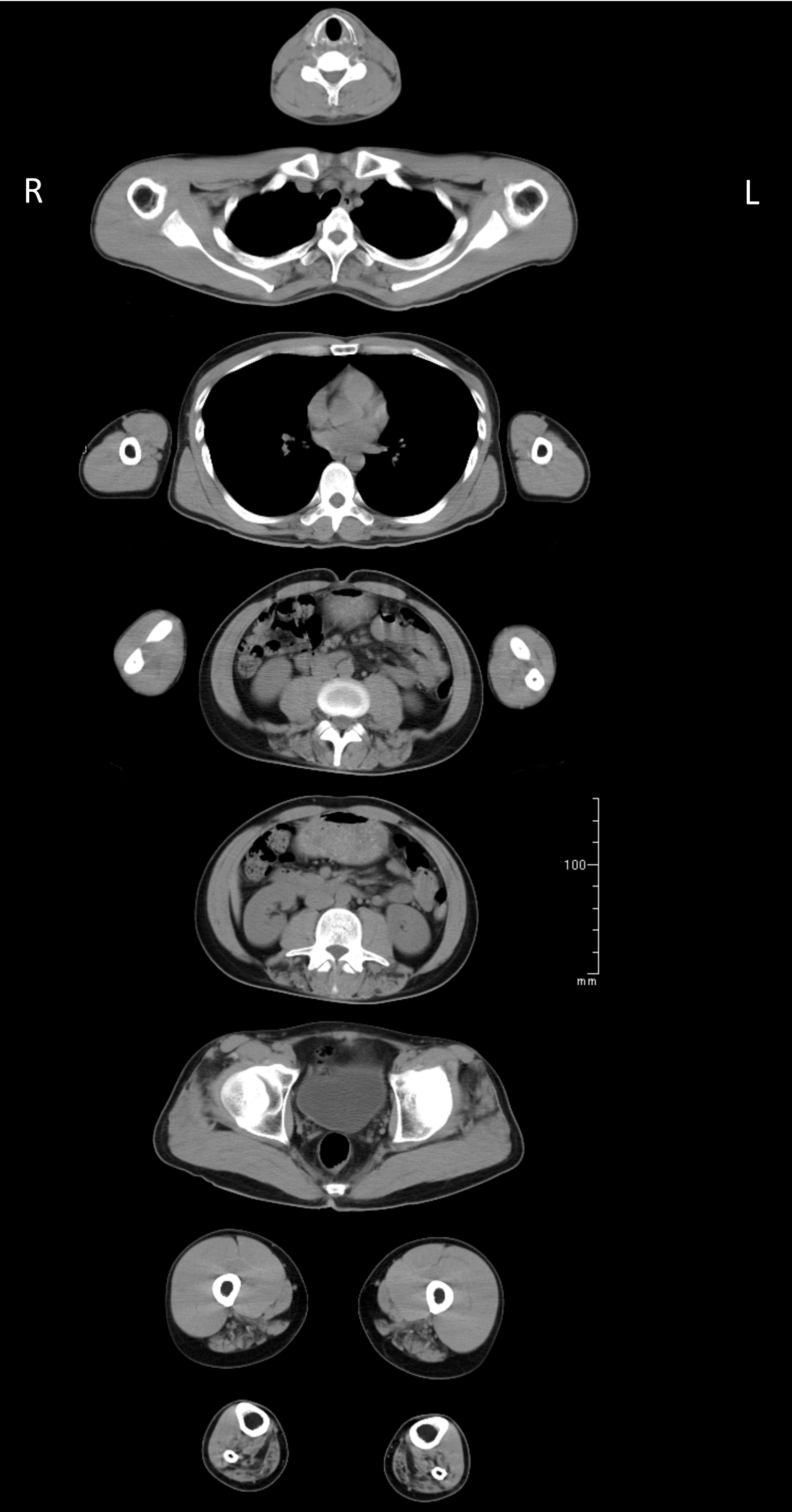

Supplement: Additional file 3: Figure S1. — Muscle CT of 29 year-old GNE myopathy patient who reported difficulty lifting his heels as one of the first symptoms. Ankle plantar flexion (MMT 2) was prominently impaired (MMT5), and muscle CT revealed that fatty replacement and atrophy were far more prominent in the calf than the anterior part of the lower legs. [file 13023_2014_150_MOESM3_ESM.tiff]
